# Data assimilation approach for addressing incompleteness in pedestrian flow measurement techniques using particle filter

**DOI:** 10.1371/journal.pone.0349624

**Published:** 2026-05-21

**Authors:** Ryo Murata, Kenji Tanaka

**Affiliations:** Department of Technology Management for Innovation, Graduate School of Engineering, The University of Tokyo, Tokyo, Japan; King Abdullah University of Science and Technology, SAUDI ARABIA

## Abstract

Understanding the dynamics of pedestrian flow in urban areas is crucial for decision-making in urban planning and marketing strategies. Previous methods for analyzing pedestrian flow can be divided into data-driven approaches and simulation-driven approaches. While data-driven approaches effectively capture actual patterns of pedestrian flow, they face the challenge of data incompleteness. On the other hand, simulation-driven approaches can generate complete data on a computer, but they only consider some of the factors determining human behavior, resulting in deviations from actual pedestrian flow. Each approach has its own limitations, yet combining them can mutually resolve these shortcomings. This paper proposes a method that applies data assimilation, a fusion technique of data-driven and simulation-driven approaches, to agent-based simulation. Combining these approaches allows for the collection of more comprehensive pedestrian flow data that better represents real-world human behavior. We conducted an evaluation experiment to assess the effectiveness of the proposed method in addressing three types of incompleteness in pedestrian flow data. The results indicate that the proposed method can effectively address data incompleteness. These findings provide guidelines for supplementing sparse measurement data in real-world environments.

## Introduction

Recent advancements in mobile devices have made it possible to observe people’s behaviors in detail. For example, GPS in smartphones can track the two-dimensional movements of people across wide areas, like entire cities. Additionally, by using Wi-Fi or Bluetooth signals, it’s possible to track three-dimensional movements in areas where GPS has limited reach, such as inside buildings or underground. The observation of human behavior in urban areas is becoming increasingly capable of covering our range of activities. This wealth of information obtained from numerous sensors can be useful in decision-making for urban planning [1] and marketing strategies [2].

However, current measurement techniques have limitations. For example, traditional camera-based measurement can only track movements within the camera’s field of view, making wide-area measurement difficult. Bluetooth/Wi-Fi-based measurement are limited to people who have installed specific apps, hindering comprehensive measurement. These incompleteness in pedestrian flow measurement methods limit the acquisition of information for understanding human behavior or lead to incorrect decisions due to biased sampling [3].

Alongside measurements, pedestrian flow simulations have been used to understand human behavior. In simulations, models are created based on variables influencing human behavior, allowing for computer-based pseudo-verification for understanding phenomena and prediction. Unlike measurements, simulations can acquire complete data as people’s movements are recorded on the computer. Especially, advancements in computing power have enabled the execution of methods like agent-based simulations, which account for the diverse behavioral characteristics of each individual. Such models can describe not just crowd behavior during events or evacuations but also everyday behaviors like city roaming [4], and purchasing activities [5]. This expands the scope of simulations to include everyday scenarios, not just specific situations where crowds form.

However, simulations alone can diverge from actual pedestrian flow, making it challenging to extract practical insight [6]. This is because pedestrian flow models extract only a portion of the factors that influence people’s behaviors, while disregarding other factors. Especially in scenarios with diversity in individual destinations, such as roaming behavior, the difference between simulation and actual pedestrian flows can become significant.

To address the incompleteness of pedestrian flow measurement and the divergence from real environments in simulation, the technique of data assimilation, rooted in earth sciences [7], can be employed. Data assimilation is a hybrid method of measurement and simulation, incorporating observational data into simulation to make accurate state estimations. It evaluates the differences between observational data and simulation while considering uncertainties, and the state of the simulation is updated to be closer to the observational data. In earth sciences, this method has been used to reasonably estimate hard-to-observe physical quantities from other observational data. Applying data assimilation to the simulation of pedestrian flow can complement the limitations of measurement techniques. This approach allows for the acquisition of comprehensive data that accurately reflects actual pedestrian flow.

This paper proposes a data assimilation method that combines agent-based simulation and particle filter to supplement the incompleteness in pedestrian flow measurement data. First, we organize the current major measurement techniques and describe the incompleteness they are facing. Then, we explain how the proposed method can complement the incompleteness. Finally, to validate our proposed method, we conduct an experiment in a virtual commercial facility, estimating the overall pedestrian flow from the individual store’s people inflow count data and comparing these estimates with pseudo-observational data.

The contributions of this paper are as follows:

- Organizing the main incompleteness in current pedestrian flow measurement techniques, and proposing an integrated method of measurement and simulation to supplement them.
- Proposing a practical data assimilation framework capable of simultaneously handling the flow of hundreds of people.
- Conducting experiments in a virtual environment and demonstrating the effectiveness of the proposed method, which outperforms conventional approaches.

## Related work

### Data-driven approach

In data-driven approaches for pedestrian flow data, machine learning is a common method. The machine learning models learn from vast datasets, identifying underlying patterns to predict outcomes and handle missing data. To capture spatio-temporal dependencies in pedestrian flow data, time series models like RNN [8], LSTM [9], and attention-based models [10–12] have been employed for both prediction and completion tasks. Additionally, generative models such as VAE [13], GAN [14], and Diffusion Models [15,16] have been applied to generate longer sequences. These models can capture complex dependencies in the data by learning from large datasets.

However, pedestrian flow data cannot always be obtained in large quantities. Currently, GPS and mobile phone tower data are widely used for collecting large amounts of pedestrian flow data. GPS does not work well indoors as the signals are often blocked [1]. Phone tower data can comprehensively measure location both indoors and outdoors; however, its accuracy is limited to a few hundred meters, restricting its usability in certain contexts. Pedestrian flow of indoor environment can be measured using sensors like cameras, Bluetooth/Wi-Fi, and LiDAR [17]. However, these methods have limitations, such as limited range or the requirement to install specific applications, making it challenging to collect large-scale and extensive data. These technical limitations make it challenging to apply machine learning models that rely on large datasets to predict pedestrian flow data, particularly in indoor environments where data is often missing.

In this paper, we propose a method for addressing **incompleteness in** pedestrian flow data using sensors such as cameras, Bluetooth, or Wi-Fi, which can be used both indoors and outdoors, without requiring pre-training on large datasets.

### Agent-based model for pedestrian flow simulation

Agent-based simulation is a bottom-up approach for revealing the mechanisms of macro phenomena like pedestrian flow by defining the behavior rules and interactions of agents [18]. Even with simple behavioral rules for each agent, the accumulation of probabilistic behavior choices and interactions allows for simulating complex systems.

Pedestrian flow models are broadly divided into macro models, which view pedestrian flow as a fluid to describe overall behavior, and micro models, which describe the behavior of individual people [19]. The agent-based model is classified as a micro model, capable of describing pedestrian flow considering differences in individual behavioral characteristics.

Agent-based models often describe pedestrian flow in specific scenarios such as evacuation [20] and infection spread [21]. In such cases, models like the Social Force Model [22], which focus on behaviors that maintain a certain distance while moving towards a destination, are commonly used. While these models are effective in scenarios involving high-density crowds and limited destinations, they are not as well-suited for situations with a variety of destinations and paths, such as roaming behaviors.

Examples of behavior models include models describing roaming behavior (movements where individuals explore a space without a fixed destination or pre-determined path), purchasing behavior [5,23], and everyday movements in urban areas [24]. This paper proposes a data assimilation method applicable to roaming behavior models with significant differences in individual behaviors. Since roaming behavior is diverse, it is challenging to accurately describe its dynamics solely with models. Therefore, by combining the data assimilation method with agent-based simulation, we aim to improve the accuracy of pedestrian flow estimation.

### Agent-based simulations with data assimilation

Applying data assimilation to agent-based simulation enables high-accuracy simulation of real-world pedestrian flow. The Kalman Filter, a standard method in data assimilation, is applicable to linear, Gaussian state transition models [25]. However, for rule-based models like agent-based models that determine individual state transitions, an analytical state description is challenging, so the Kalman Filter itself is not directly usable. Extensions of the Kalman Filter include the ensemble Kalman filter [26,27] and the unscented Kalman filter [28], applicable to nonlinear models. These methods can perform data assimilation for nonlinear state transition models with relatively low computational cost, but they require Gaussian assumptions in state transition models, limiting their application.

We note that agent-based models do not necessarily always produce non-Gaussian state distributions, and that nonlinearity alone does not immediately imply non-Gaussianity. However, in the agent-based model considered in this study, each agent makes probabilistic and history-dependent decisions, and these outcomes are aggregated across multiple agents. In such aggregation processes, multiple behavioral patterns can coexist simultaneously, resulting in posterior distributions of states and observables that are asymmetric and multi-modal. Therefore, approximating the state distribution with a single Gaussian distribution is statistically inappropriate, and Kalman Filter-based methods would lead to degraded estimation accuracy. The particle filter, which uses sample-based approximation, can flexibly represent such non-linear and non-Gaussian state transition models, whereas EnKF and UKF rely on Gaussian assumptions and thus cannot adequately capture multi-modal distributions.

The particle filter, which estimates the system’s state distribution using a set of weighted samples (particles), is particularly suitable for nonlinear and non-Gaussian state transition models. Previous studies that have used particle filters in agent-based simulations include those that improve route selection parameters in evacuation behavior [29] or those integrating data from multiple sensors at live events [30]. These are examples of describing the flow of people when crowds move in a unified manner.

For pedestrian flows with significant differences in individual behaviors, such as roaming, examples include simulations in a train station [31,32] or a single floor of a building [33]. These focus on real-time operation, targeting parts of facilities or very few individuals. While the method proposed in this study also has potential for real-time estimation applications, we use particle filter to address the incompleteness of pedestrian flow measurement techniques. Therefore, we propose a practical data assimilation method for scenarios where a large number of people roam in extensive spaces like entire facilities.

## Data assimilation framework

### Particle filter

The particle filter is a data assimilation method that approximates the system model using Monte Carlo sampling [34]. It constructs a distribution based on a group of samples extracted from models, making it applicable to non-linear and non-Gaussian state-space models. This flexibility allows it to be applied to complex phenomena resulting from interactions between agents, as seen in agent-based simulations.

The primary goal of a particle filter is to estimate the current state of the system based on accumulated observational data up to the present. Let us consider the situation where the state at time *t* − 1 is estimated based on observational data up to time *t* − 1. The state at time *t* − 1 can be represented as follows:


$$\begin{array}{c} x_{t-1}\sim p(x_{t-1}|y_{1:t-1}) \end{array}$$ (1)

where *x*_*t*−1_ represents the state at time *t* − 1 and $y_{1:t-1}$ represents the measurement from time 1 to *t* − 1. Next, the state at time *t* is estimated based on the observations at time *t* − 1 as follows:


$$\begin{array}{c} p(x_{t}|y_{1:t-1})=\frac{1}{N}\sum_{i=1}^{N}\delta (x_{t}-x_{t|t-1}^{\left(i\right)}) \end{array}$$ (2)

where δ represents the Dirac delta function, and *N* is the total number of particles. $x_{t|t-1}^{\left(i\right)}$ is the state of the i-th particle estimated by the transition model. This expression implies that the state at time *t* is approximated by an ensemble of *N* particles based on the observational data up to time *t* − 1.

Then, the state estimation at time *t* is performed based on the observations obtained at time *t*. This can be represented as follows:


$$\begin{array}{c} p(x_{t}|y_{1:t})=\sum_{i=1}^{N}w_{t}^{\left(i\right)}\delta (x_{t}-x_{t|t-1}^{\left(i\right)}) \end{array}$$ (3)

where $w_{t}^{\left(i\right)}$ represents the weight of particle *i*. During the resampling process, particles are generated in accordance with these weights. The weight $w_{t}^{\left(i\right)}$ is calculated as follows:


$$\begin{array}{c} w_{t}^{\left(i\right)}=\frac{p(y_{t}|x_{t|t-1}^{\left(i\right)})}{\sum_{j}p(y_{t}|x_{t|t-1}^{\left(j\right)})} \end{array}$$ (4)

where $p(y_{t}|x_{t|t-1}^{\left(i\right)})$ represents the probability distribution of the observational value given the state, which is known as the likelihood. Likelihood indicates how well each particle explains the observational data. By selecting particles based on likelihood, particles closer to the observational data are likely selected.

**Fig 1:**
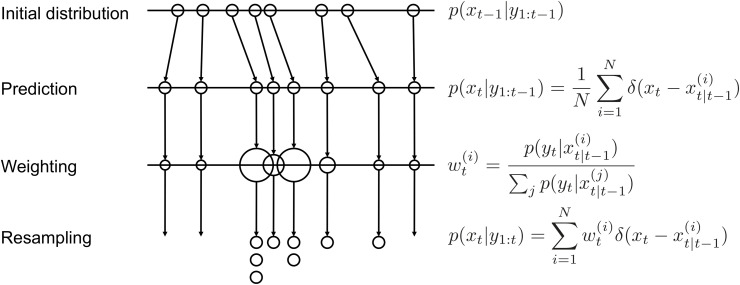
The basic process of particle filtering. Initially, a large number of particles are generated for each entity, with the model projecting the state for the next step. The weight of each particle is determined by the likelihood calculated from observational data. A resampling process based on this likelihood is then conducted to establish the distribution of the predicted state for the next step.

Fig 1 shows a diagram of a particle filter process. By state prediction through the model and resampling weighted by likelihood, the simulation can estimate the system state accurately.

### Integrating agent-based simulation with particle filters

When incorporating a particle filter into agent-based simulation, the key considerations are 1) defining the state variables and 2) defining the observation variables and likelihood. The state variables should be chosen to characterize roaming behavior. Observation variables should be defined as those that provide valuable information for estimating actual state. Likelihood should be defined in a way that reduces the uncertainty of the simulation based on the observation variables. By considering these aspects, we can incorporate real-world roaming tendencies into agent-based simulation via particle filtering.

In our implementation, we apply a particle filter independently to each agent. Specifically, each agent maintains its own set of particles that represent candidate next-store locations. Each particle in this set corresponds to a potential state (destination store) for that specific agent, rather than representing the entire model state across all agents. This approach differs from implementations where particles represent the full system state (e.g., [31,32]), where a single particle ensemble tracks all agents simultaneously. We adopt this per-agent filtering approach because it allows us to align individual agent behaviors with real-world observations in a bottom-up manner, which in turn enables the reproduction of statistical properties at the aggregate level.

Firstly, the state variable is defined as each agent’s location. This is because comprehensive measurement of agent’s location is difficult, and the information is important for characterizing the roaming behavior. To estimate the next step locations of agents, particles are generated for each agent based on models. These particles stochastically determine the destination.

Observation variables are chosen as the inflow count data at each point. This is because by sequentially measuring the inflow data at each location, it is possible to calculate the likelihood based on the real-world visitation tendencies. In other words, by using inflow count data to determine which stores are more or less likely to be visited, it is possible to align the roaming tendencies of the agents more closely with those in the real world. This allows for the transition of agents based on transition models and some measured data to reflect real pedestrian flow tendencies.

Based on these methods, the simulation proceeds as follows:

1. Select an agent and perform state transitions for the number of particles.
2. Calculate the likelihood of each particle.
3. Normalize the likelihoods to convert them into a probability distribution.
4. Select one particle from the weighted particles as the agent’s location for the next step. The selection is performed by sampling according to the normalized weights (i.e., weighted random sampling), not by choosing the particle with the highest weight.

**Fig 2:**
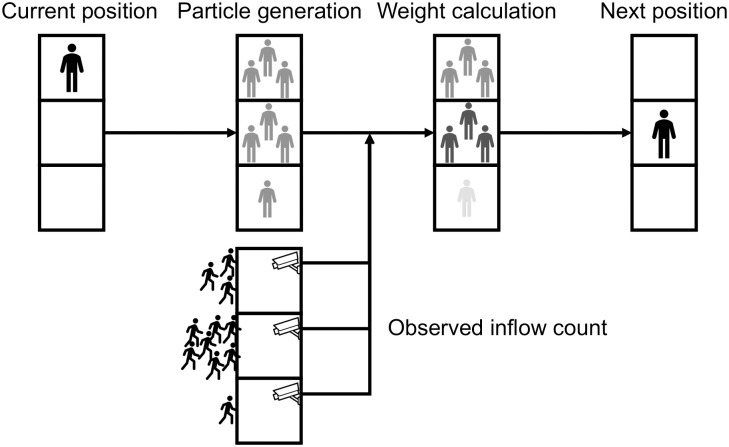
Integration of particle filters into agent-based simulation. After particles are generated by the model, they are weighted based on camera-captured inflow data. Agents move to locations that are most likely to be visited at time t in both the model and the real world.

Resampling is not performed in this paper because filtering is applied to each agent individually, and the number of transitions per agent is limited to only a few. It is known that in systems with a small number of transitions, skipping resampling can enhance accuracy [35]. Performing these four steps for all agents bridges the gap between the simulation and real-world pedestrian flow (Fig 2).

### Addressing the incompleteness of pedestrian flow data

**Fig 3:**
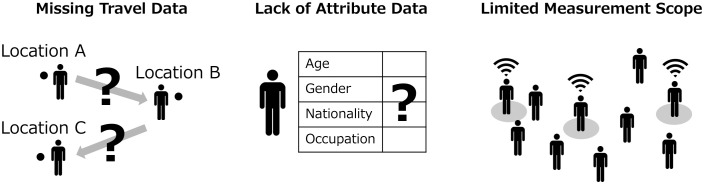
Three types of incompleteness in current people flow measurement techniques.

As mentioned in related works, incompleteness of pedestrian flow data can be classified into three categories. Below, we discuss guidelines for data assimilation to address these three types of incompleteness (Fig 3).

To characterize these three types of incompleteness in a unified manner, we introduce the following notation. Let *X* denote the location space to which individual locations belong; *X* can be discrete or continuous. For an individual *i* at time step *t*, let $x_{i}(t)\in X$ denote the location and **a**_*i*_ denote the time-invariant attributes (e.g., age, gender). We further introduce an observation indicator


$$\begin{array}{c} o_{i}(t)=\left\{ \begin{array}{cc} 1 & \text{if }x_{i}(t)\text{ is observed,}\\ 0 & \text{otherwise,} \end{array}\right. \end{array}$$ (5)

and define the partially observed trajectory of individual *i* as


$$\begin{array}{c} {𝐬}_{i}=\{(t,x_{i}(t)):t\in \{1,\ldots ,T\},\;o_{i}(t)=1\}, \end{array}$$ (6)

where *T* is the maximum time step. A perfectly recorded trajectory corresponds to the case in which *o*_*i*_(*t*) = 1 for all t∈{1,…,T} together with fully known **a**_*i*_. The three types of incompleteness described below correspond to different patterns of missingness in *o*_*i*_(*t*) and **a**_*i*_.

#### Missing travel data.

When the measurement range of sensors is limited, it can lead to the absence of comprehensive travel data, including detailed point-to-point transition information. This issue is particularly prevalent with devices like cameras or LiDAR, which hinders the collection of continuous OD data across entire areas.

Using the notation introduced above, this type of incompleteness corresponds to the case in which *o*_*i*_(*t*) = 0 for some time steps; that is, location is only intermittently observed. The partially observed trajectory **s**_*i*_ therefore contains only those time steps at which *o*_*i*_(*t*) = 1, and the transitions between unobserved time steps remain unknown, preventing the reconstruction of complete trajectories.

To address the missing travel data, we adopt a method that links localized count data, such as from cameras, to agent-based simulations. The approach involves determining the likelihood of an agent’s next destination based on visitation tendencies. As outlined in the preceding section, this method utilizes inflow count data from each location to incorporate real-world visitation tendencies to the simulation. By doing this, we can get continuous travel data through discrete sensor data.

#### Lack of attribute data.

Lack of attribute data refers to the inability to link attribute information with travel data. This limitation is common in many sensors, with the exception of GPS devices. In roaming behavior, attribute information, which clarifies behavioral characteristics, is essential for conducting a detailed analysis.

Using the notation introduced above, this incompleteness corresponds to the case in which the attribute vector **a**_*i*_ is unknown, preventing the linking of behavioral characteristics to movement patterns.

To address the lack of attribute data, it is effective to apply attribute information to agent-based simulations. Currently, cameras serve as the primary means of capturing attribute information such as age and gender in both indoor and outdoor settings. However, they fall short in tracking comprehensive movements, such as transitions between points. For estimating transition data based on attributes using cameras, it is appropriate to gather inflow count data for each attribute and estimate the transition tendencies. This approach applies the method discussed in the previous subsection to each attribute identified by cameras. This enables the linking of movement data with attribute information such as age and gender. In addition, as a secondary effect, it can improve the system’s accuracy by reducing estimation uncertainty through attribute-based estimates.

#### Limited measurement scope.

A limited measurement scope restricts the ability to observe everyone in a target area, a common issue with sensors like Bluetooth/WiFi. While these sensors can capture continuous transition sequence, they risk biased sampling, leading to inaccurate estimations. For example, consider a scenario where individuals with attribute “A” have a higher rate of smartphone ownership and often opt for transition sequence “a”, while those with attribute “B” have a lower rate of smartphone ownership and tend to prefer transition sequence “b”. In this scenario, using Bluetooth/WiFi detection would likely result in a higher detection rate of individuals with attribute “A” due to their higher smartphone ownership. This could lead to an overestimation of transition sequence “a” and an underestimation of transition sequence “b”. As this example indicates, limiting measurement scope to only a subset can lead to biased estimations.

Using the notation introduced above, this incompleteness corresponds to the case in which only a biased subset 𝒮⊂{1,2,…,N} of the total population *N* is observable; that is, *o*_*i*_(*t*) = 0 for all t∈{1,…,T} when i∉𝒮. The sampling bias $\mathrm{Pr}(i\in 𝒮|{𝐚}_{i})$ depends on attributes, leading to skewed observations.

To address the limited measurement scope, it is effective to modify the recorded sequences using inflow count data. The reason is that count data can reveal the visitation tendency of each location, allowing for an indirect estimation of the frequency of occurrence of transition sequences. Transition sequences including locations with a higher visitation rate can be assumed to have a higher frequency of occurrence, while those with a lower visitation rate can be assumed to have a lower frequency. By adjusting the frequency of occurrence of transition sequences based on the weighting of count data, it is possible to mitigate the bias caused by sampling.

## Experiment

We evaluate the efficacy of a data assimilation method that integrates agent-based simulation and particle filtering. This method is designed to tackle the three types of incompleteness in pedestrian flow measurement techniques highlighted in the previous section. We estimate the transition tendencies of pseudo-observational data by applying a particle filter. The simulation setting involves a virtual commercial facility, which is used to simulate the shopping behavior of customers.

### Model

The proposed method can be applied to any agent-based model. For this experiment, we use a simplified version of the purchasing behavior model proposed by Hui et al [36]. This model has been validated for its reliability based on trajectory data of thousands of customers.

#### Model environment.

The simulation is conducted in a virtual commercial facility with 18 stores. In the implementation, the commercial facility is represented as a graph, with each store depicted as a node. All nodes of the stores are interconnected, allowing each agent to move freely between facilities. We only record the transitions between stores, without considering the specific routes taken.

#### Agent dynamics.

The agent dynamics are governed by the following formula:


$$\begin{array}{c} P_{i,j}=\frac{\exp\left[k_{i}\left(A_{j}+\sum_{j^{\prime }\neq j}\frac{A_{j^{\prime }}}{(1+d_{j,j^{\prime }}{)}^{\lambda }}\right)+\omega_{i}\rho_{j,t}\right]}{\sum_{j}\exp\left[k_{i}\left(A_{j}+\sum_{j^{\prime }\neq j}\frac{A_{j^{\prime }}}{(1+d_{j,j^{\prime }}{)}^{\lambda }}\right)+\omega_{i}\rho_{j,t}\right]} \end{array}$$ (7)

where $P_{i,j}$ is the probability of agent *i* visiting store *j*, *A*_*j*_ is the unique attractiveness of store *j*, $d_{j,j^{\prime }}$ is the distance between stores *j* and $j^{\prime }$, $\rho_{j,t}$ is the congestion level of store *j* at time *t* (defined as the current number of agents visiting store *j* at time *t*), and ω, *k*, and λ are coefficients. This model indicates that the probability of visiting a store is determined by its unique a*tt*ractiveness, the attractiveness of surrounding stores, and the s*t*ore’s congestion level. The congestion term $\rho_{j,t}$ represents the primary form of agent-agent interaction in the model.

### Experimental condition

#### Environment.

To assess the effectiveness of data assimilation using particle filtering, we conducted tests in three distinct scenarios. These tests aim to evaluate if our method can effectively address the incompleteness in pedestrian flow measurement techniques. Initially, we outline the conditions that are consistent across all scenarios.

We initiated the simulation with 100 agents, assigning them random starting stores. Each agent spends 2–3 steps in a store before moving on to the next. After making this transition between stores three times, the agents stop roaming. Once the number of stationary agents reaches 40, we introduce an additional 40 agents to replace them. This process of replenishment continues, simulating the roaming patterns of a total of 2000 agents over 200 steps. We ran the simulations thirty times and averaged the results to obtain the final outcome.

We divided the 2000 agents into four distinct groups, with 500 in each group, all exhibiting unique behavioral patterns. These patterns are influenced by how attractive each store is. We have set parameter *A* to different levels based on the store and its corresponding group: 7.5 for stores 0–2 in group 1, 8 for stores 3–5 in group 2, 8.5 for stores 6–8 in group 3, and 10 for stores 9–11 in group 4. All other stores in each group default to a value of 5. The values of the remaining parameters ω, *k*, and λ are set to 0.005, 1, and 6, respectively. These values were chosen by referring to the parameter settings reported in [36] and by empirically tuning them so that the diversity of shopping behaviors observed in the simulated trajectories is maintained across agents.

The number of particles in the particle filter was set to 100 for Cases 1 and 2, and 400 for Case 3. These values were determined to be sufficient for the experimental environment, considering the diversity of transition sequences in each case. The assimilation frequency was set to 3 steps, which was empirically determined from the perspective of estimation accuracy. For the EnKF used in Case 3 for comparison, the ensemble size was set to 400, matching the number of particles in the particle filter to ensure a fair comparison.

#### Observational data.

In a similar manner to previous works [29–31,33], the performance of the data assimilation framework was evaluated by the identical twin experiment. In the identical twin experiment, observational data is generated from the model itself, and data assimilation is performed based on the pseudo-observational data. The data assimilation framework is then evaluated by comparing the pseudo-observational data with the estimates from the data assimilation.

To properly validate the effectiveness of the data assimilation method in twin experiments, we change the values of the simulation parameters from those used in the data assimilation model environment. In this experiment, the attractiveness of each store, represented by *A*, is varied between both environments. Within the pseudo-observational model environment (ground-truth), parameters are set as described in the previous subsection. For the data assimilation model environment, all parameters are uniformly set to 5. This approach tests the efficacy of our proposed method in scenarios where information about the relative attractiveness of each store is not available.

#### Benchmark methods.

In Case 3, we compare our proposed particle filter method with three baseline approaches:

*Baseline*: This method performs probabilistic sampling from the observed transition sequence distribution. Specifically, for each new agent, a three-store transition sequence is randomly selected from the distribution of sequences observed in the pseudo-observational data, weighted by their observed frequencies.

*VAE (Variational Autoencoder)*: We train a conditional VAE on the three-store transition sequences extracted from the observed data. The VAE learns to encode and decode these sequences, and during estimation, we sample new sequences from the learned latent space. The input to the VAE is a three-store sequence (*s*_1_, *s*_2_, *s*_3_), and the output is a reconstructed sequence that respects the constraint that stores cannot be revisited.

*EnKF (Ensemble Kalman Filter)*: We apply the Ensemble Kalman Filter to estimate the attractiveness parameters *A*_*j*_ for each store. The state vector consists of the attractiveness values for all stores, and the observations are the inflow count data. The EnKF maintains an ensemble of state vectors and updates them based on the observed inflow counts. The estimated attractiveness values are then used in the transition probability model (Equation 1) to determine agent movements.

#### Simulation case.

The experiment is conducted under three different cases using particle filters for data assimilation. These cases correspond to the three types of incompleteness identified earlier: Case 1 addresses missing travel data, Case 2 addresses lack of attribute data, and Case 3 addresses limited measurement scope.

*Case 1 — Count Data Available at Each Store (addressing missing travel data)*

In this case, we estimate the transition tendencies from the observed data using the time-series inflow count data for each store. This case corresponds to the “missing travel data” incompleteness, where continuous point-to-point transition information is unavailable. The sensors assumed in this setup are cameras and LiDAR. We calculate the likelihood of an agent moving to a specific store using the formula below:


$$\begin{array}{c} w_{\text{store}}^{\left(i\right)}(t)*=\exp(inflow_{\text{obs}}^{\left(i\right)}(t)) \end{array}$$ (8)

where $inflow_{\text{obs}}^{\left(i\right)}(t)$ represent the number of agents entering store *i* at step *t*. Updating the likelihood in this way enables *t*he simulation to accurately mirror the visiting tendencies in the real world. The number of particles is set at 100. The resampling procedure is summarized in Algorithm 1.

**Algorithm 1 Filtering process using inflow count data**

**Input:** Observed inflow count data $inflow_{\text{obs}}^{\left(i\right)}(t)$ for each store *i* at time *t*, number of stores *N*_*store*_

**procedure** Filtering process

**for** *t* = 1 to *T* **do**

**for** *i* = 1 to *N*_*store*_ **do**

${\tilde{w}}_{store}^{\left(i\right)}(t)*=\exp(inflow_{\text{obs}}^{\left(i\right)}(t))$    ▷ Update unnormalized weights

**end for**

$w_{store}^{\left(i\right)}(t)=\frac{\overset{\left(i\right)}{\underset{store}{\tilde{w}}}(t)}{\sum_{j=1}^{N_{store}}{\tilde{w}}_{store}^{\left(j\right)}(t)}$         ▷ Normalize weights across all stores

**for all** i∈New_agent **do**

$Store(i)=\text{select}(w_{store}(t))$     ▷ Select store by weighted random sampling

**end for**

**end for**

**end procedure**

**Output:** Selected store for each new agent at each time step

*Case 2 — Count Data with Attributes Available (addressing lack of attribute data)*

In this case, we estimate the transition tendencies with attribute from the observed data using the time-series attribute tagged inflow count data for each store. This case corresponds to the “lack of attribute data” incompleteness, where attribute information cannot be linked with travel data. The sensors assumed in this setup are cameras, which can capture attribute information such as age and gender. In this experiment, attributes represent four mutually exclusive categorical groups (e.g., age bands: 20s, 30s, 40s, 50+), each with distinct store preferences. We calculate the likelihood of an agent moving to a specific store using the formula below:


$$\begin{array}{c} w_{store}^{\left(i\right)}(attr,t)*=\exp(inflow_{\text{obs}}^{\left(i\right)}(attr,t)) \end{array}$$ (9)

where $inflow_{\text{obs}}^{\left(i\right)}(attr,t)$ represents the number of agents with the attribute *attr* entering store *i* at step *t*. The calculation of likelihood for each attribu*t*e follows the same formula as in Case 1. The number of particles is set at 100. The resampling procedure is summarized in Algorithm 2.

**Algorithm 2 Filtering process using inflow count data with attribute information**

**Input:** Attribute-tagged inflow count data $inflow_{\text{obs}}^{\left(i\right)}(attr,t)$ for each store *i* and attribute *attr* at time *t*, number of stores *N*_*store*_, number of attribu*t*es *N*_*attr*_

**procedure** Filtering process

**for** *t* = 1 to *T* **do**

**for** attr = 1 to *N*_*attr*_ **do**

**for** *i* = 1 to *N*_*store*_ **do**

${\tilde{w}}_{store}^{\left(i\right)}(attr,t)*=\exp(inflow_{\text{obs}}^{\left(i\right)}(attr,t))$  ▷ Update unnormalized weights for each attribute

**end for**

$w_{store}^{\left(i\right)}(attr,t)=\frac{{\tilde{w}}_{store}^{\left(i\right)}(attr,t)}{\sum_{j=1}^{N_{store}}{\tilde{w}}_{store}^{\left(j\right)}(attr,t)}$  ▷ Normalize weights across stores for each attribute

**for all** *i* in New_agent with attribute *attr* **do**

$Store(attr,i)=\text{select}(w_{store}(attr,t))$   ▷ Select store by weighted random sampling

**end for**

**end for**

**end for**

**end procedure**

**Output:** Selected store for each new agent (with corresponding attribute) at each time step

*Case 3 — Count data and partial transition sequence data available (addressing limited measurement scope)*

In this case, we estimate the transition tendencies using the time-series inflow count data for each store and biased samples of transition sequence. This case corresponds to the “limited measurement scope” incompleteness, where only a subset of individuals can be observed, leading to biased sampling. The sensors assumed in this setup are Bluetooth/Wi-Fi and RFID. Differing from previous cases where particles were solely model-generated, here we utilize the measured transition sequence samples as the particles. This is because using samples of real-world transition sequences allows for the replication of longer overall transition tendencies. We calculate the likelihood of the transition sequences using the formula below:


$$\begin{array}{c} w_{store}^{\left(i\right)}(t)*=\exp(inflow_{\text{obs}}^{\left(i\right)}(t)) \end{array}$$ (10)

$$\begin{array}{c} w_{sequence}^{\left(i\right)}(t)=\sum_{k\in sequence(i)}w_{store}^{\left(k\right)}(t) \end{array}$$ (11)

where $w_{store}^{\left(k\right)}(t)$ represents the weight of the transition sequence *k*. This implies that sequences including stores with high inflow counts are more likely to be selected. In this case, we assume that the samples are biased and consider a scenario where agents with four different attributes are sampled at respective ratios of 0.4, 0.25, 0.2, and 0.15. To evaluate the effectiveness of our proposed method under conditions of biased sampling, we compare the accuracy of selecting sequences randomly versus selecting them based on the likelihood. The number of particles is set at 400. The resampling procedure is summarized in Algorithm 3.

Furthermore, in Case 3, we conducted comparative experiments with conventional approaches, including a baseline method based on probabilistic sampling from the sequence distribution, a Variational Autoencoder (VAE) [37], and the Ensemble Kalman Filter (EnKF) [38].

**Algorithm 3 Filtering process using inflow count data and partial transition sequence data**

**Input:** Observed inflow count data $inflow_{\text{obs}}^{\left(i\right)}(t)$ for each store *i* at time *t*, sampled transition sequences, number of stores *N*_*store*_, number of sequences *N*_*sequence*_

**procedure** Filtering process

**for** *t* = 1 to *T* **do**

**for** *i* = 1 to *N*_*store*_ **do**

${\tilde{w}}_{store}^{\left(i\right)}(t)*=\exp(inflow_{\text{obs}}^{\left(i\right)}(t))$  ▷ Update unnormalized store weights

**end for**

$w_{store}^{\left(i\right)}(t)=\frac{{\tilde{w}}_{store}^{\left(i\right)}(t)}{\sum_{j=1}^{N_{store}}{\tilde{w}}_{store}^{\left(j\right)}(t)}$   ▷ Normalize store weights

**for** *i* = 1 to *N*_*sequence*_ **do**

${\tilde{w}}_{sequence}^{\left(i\right)}(t)=\sum_{k\in sequence(i)}w_{store}^{\left(k\right)}(t)$   ▷ Compute sequence weight as sum of constituent store weights

**end for**

$w_{sequence}^{\left(i\right)}(t)=\frac{{\tilde{w}}_{sequence}^{\left(i\right)}(t)}{\sum_{j=1}^{N_{sequence}}{\tilde{w}}_{sequence}^{\left(j\right)}(t)}$   ▷ Normalize sequence weights

**for** i∈New_agent **do**

$Sequence^{\left(i\right)}(t)=\text{select}(w_{sequence}(t))$  ▷ Select sequence by weighted random sampling

**end for**

**end for**

**end procedure**

**Output:** Selected transition sequence for each new agent at each time step

## Results and discussion

**Fig 4:**
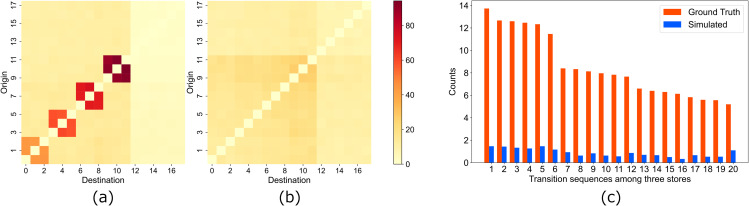
Comparison of ground truth and simulated transition tendencies in case 1. **(a)** Ground truth values of the OD (Origin-Destination) matrix. The axis values represent store numbers, with the vertical axis indicating the store of departure and the horizontal axis indicating the arrival store. **(b)** Estimated values based on data assimilation. The axis values represent store numbers, with the vertical axis indicating the store of departure and the horizontal axis indicating the arrival store. **(c)** Comparison of the top 20 most frequent three-store transition sequences from ground truth and estimated. The horizontal axis numbers represent the ranking of transition frequency, and the vertical axis shows their frequencies.

Fig 4 (a) and 4 (b) compare the OD matrix from the observed environment with those from the data assimilation environment in Case 1. It is clear that estimating pedestrian movement tendencies between stores based solely on inflow count data is not effective. Fig 4 (c) presents a comparison of the transition tendencies among three stores, further indicating that the proposed method falls short in estimation accuracy. This difficulty arises from the uncertainty associated with inflow count data, which does not allow for individual identification. As this experiment assumes a variety of roaming behaviors, many stores exhibit high inflow counts, resulting in a wide range of transit options for them. This uncertainty leads to imprecise estimations.

**Fig 5:**
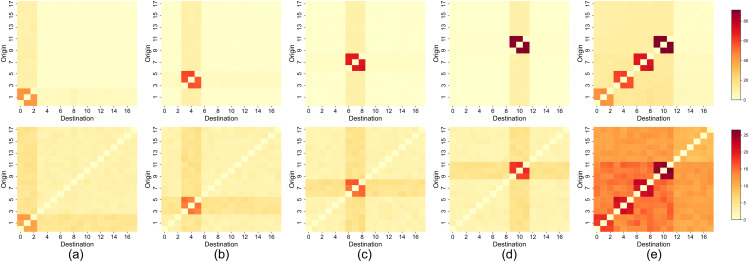
Comparison of the ground truth OD matrices (upper) and the simulated OD matrices (lower) for each attribute, along with the combined OD matrix, in Case 2. **(a)** OD matrix for attribute 1 (ground truth, upper; simulated, lower). **(b)** OD matrix for attribute 2 (ground truth, upper; simulated, lower). **(c)** OD matrix for attribute 3 (ground truth, upper; simulated, lower). **(d)** OD matrix for attribute 4 (ground truth, upper; simulated, lower). **(e)** Combined OD matrix (ground truth, upper; simulated, lower). The OD matrices from (a) to (d) are derived from data assimilation, utilizing inflow count data that can distinguish between attributes. The color bar represents the transition count between stores.

Fig 5 compares the OD matrices from the observed environment with those from the data assimilation environment for each attribute in Case 2. The transition tendencies of each attribute are relatively well replicated. This improved accuracy results from the addition of attribute information, which reduces uncertainty of estimation. However, this scenario presumes an ideal situation where agents’ behavioral characteristics are categorized by attributes. In reality, individuals sharing the same attribute often display diverse behaviors, which could increase uncertainty. The effectiveness of this method in real-world observations will need to be verified in future work.

The combined OD matrix (case (e) in Fig 5) shows some differences between observed and simulated values. In this simulation case, we estimate continuous transition data between locations from highly spatially localized inflow count data. This inherent uncertainty in the estimation process contributes to the differences observed in the OD matrices. While the matrices for attributes (a) through (d) successfully capture relative transition frequencies, they do not achieve quantitative accuracy. The combined matrix (e), which aggregates these individual matrices, accumulates the estimation errors from (a) through (d), resulting in a more dispersed distribution compared to the ground truth. Nevertheless, the system is sufficiently functional for relative discussions about which location pairs exhibit higher transition frequencies.

**Fig 6:**
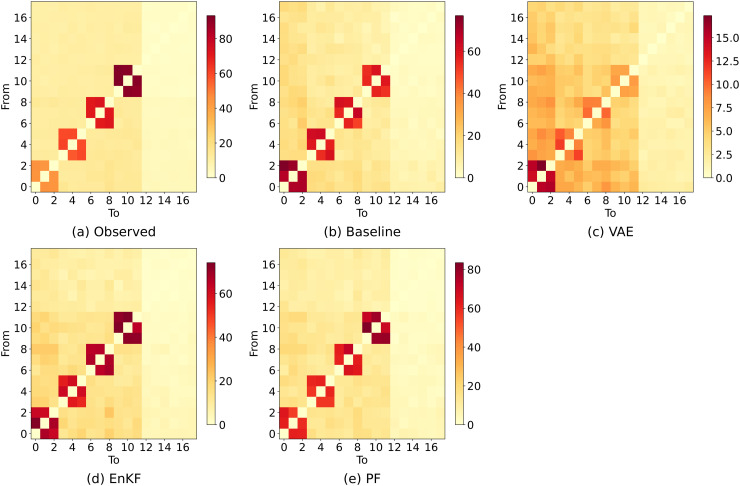
Ground truth OD matrix and estimated OD matrices for different methods in case 3. **(a)** Ground truth OD matrix. **(b)** Estimated OD matrix for baseline sampling. **(c)** Estimated OD matrix for VAE. **(d)** Estimated OD matrix for ensemble Kalman filter. **(e)** Estimated OD matrix for particle filter. The color bar represents the transition count between stores.

Fig 6 compares the OD matrices from the observed environment (a) with those obtained from the VAE (c), the EnKF (d), and our proposed particle filter–based assimilation (e). While all approaches partially capture transition tendencies, quantitative evaluation reveals clear differences across methods.

The evaluation metrics are defined as follows. The absolute error (Abs Err) is calculated as:


$$\begin{array}{c} \text{Abs Err}=\sum_{i=1}^{N_{store}}\sum_{j=1}^{N_{store}}|O_{i,j}-E_{i,j}| \end{array}$$ (12)

where $O_{i,j}$ is the observed transition count from store *i* to store *j*, and $E_{i,j}$ is the estimated transition count.

The Jensen-Shannon divergence (JSD) is calculated as:


$$\begin{array}{c} \text{JSD}(P||Q)=\sqrt{\frac{1}{2}\text{KL}(P||M)+\frac{1}{2}\text{KL}(Q||M)} \end{array}$$ (13)

where $M=\frac{1}{2}(P+Q)$, *P* and *Q* are the normalized probability distributions of observed and estimated OD matrices, respectively, and KL is the Kullback-Leibler divergence.

Recall@*k* (also denoted as TOP@*k*) measures the recall of observed transitions within the top *k* predicted sequences, where higher values indicate better ranking performance.

Table 1 shows that our proposed method achieves the lowest absolute error and the lowest Jensen–Shannon divergence, indicating the closest alignment with the observed environment. Our particle filter method also attains the highest recall values, confirming its superior ability to reproduce the most frequent transition patterns.

**Table 1:** Quantitative comparison of estimation methods in Case 3. The table shows absolute error (raw transition counts), Jensen–Shannon divergence (JSD), and Recall@K (K = 5, 10, 20). Lower error and JS divergence, and higher Recall@K, indicate closer agreement with observations.

Method	Abs Err	JSD	Recall@5	Recall@10	Recall@20
Baseline	1080.4	0.300	0.147	0.337	0.760
VAE	2743.4	0.294	0.053	0.167	0.395
EnKF	847.1	0.286	0.333	0.467	0.792
PF (Ours)	684.1	0.256	0.460	0.563	0.808

**Fig 7:**
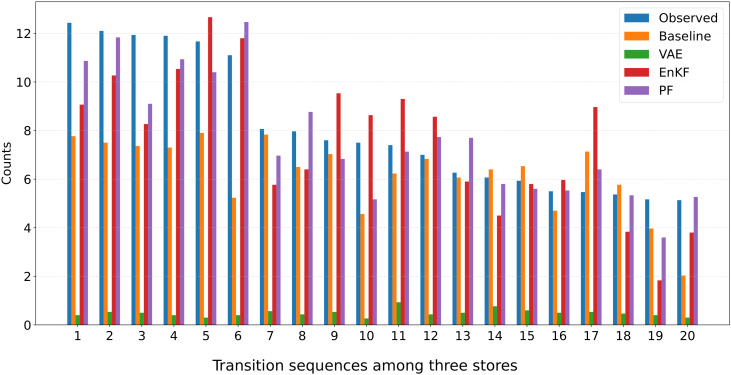
Comparison of the frequency of three-store transition sequences in Case 3. The figure shows the average counts of the top-20 transition sequences (among three stores) from ground truth and estimated by each method (Baseline, VAE, EnKF, PF). The horizontal axis corresponds to the rank of transition sequences in the ground truth data, and the vertical axis shows the average frequency across iterations.

To further examine predictive performance for longer transition structures, Fig 7 presents a bar plot of the top-20 three-store transition sequences. This analysis evaluates not only direct OD pairs but also higher-order movement patterns. The three-store transition sequences (e.g., store 3 → store 7 → store 4) capture longer-term behavioral patterns than simple OD matrices, providing a more comprehensive evaluation of the methods’ ability to reproduce realistic pedestrian movement patterns. The particle filter again reproduces the observed sequences most accurately, whereas EnKF and VAE show noticeable deviations.

The differences in performance arise from the inherent limitations of each approach. EnKF relies on the Gaussian assumption inherent in its update mechanism, which limits its effectiveness under the non-Gaussian transition dynamics observed here. VAE, in turn, was trained on data affected by sampling bias, which restricts its ability to generalize to the actual environment. By overcoming both the Gaussianity assumption and the sampling bias issue, our particle filter approach achieved the highest overall accuracy, demonstrating its robustness for complex, non-Gaussian transition dynamics.

The experiments in these three cases indicate the following regarding the three types of incompleteness in pedestrian flow measurement methods: First, compensating for “missing travel data” is challenging with only inflow count data; however, incorporating attribute information or partially sampled transition sequences appears effective. To address the “lack of attribute data”, using attribute-tagged inflow count data to complement transition sequences is beneficial. Lastly, to overcome the “limited measurement scope”, integrating comprehensive inflow count data proves effective in reducing sample bias.

## Limitations and future work

### Evaluation using real-world data

In this study, the observational data utilized for data assimilation were pseudo-generated from a model and did not accurately represent real-world pedestrian flow. Therefore, it is essential to assess the system’s effectiveness using actual data reflecting diverse human behaviors.

In addition, when collecting real-world data, it is important to take into account the potential impact of measurement errors. For instance, when gathering inflow count data, sensors such as cameras and LiDAR are susceptible to errors caused by issues like occlusion. Future work should focus on determining how significantly these errors influence the proposed system.

### Evaluation of the dynamic capabilities of the system

The data assimilation method implemented in this study is sequential, enabling a dynamic representation of real-world people flow by progressively incorporating observational data [39]. Understanding people flow in real-time is valuable for making decisions about congestion relief measures and digital advertising strategies. However, for real-time operation to be feasible, maintaining a low computational load is critical. Especially, the particle filter used in this research increases the computational burden as the number of particles grows, necessitating careful determination of the optimal number of particles and assimilation frequency, informed by empirical experiments.

## Conclusion

This paper proposed a data assimilation framework combining agent-based simulation and particle filter to complement the incompleteness in pedestrian flow measurement techniques. We identified three main shortcomings of existing pedestrian flow measurement techniques as “missing travel data”, “lack of attribute data”, and “limited measurement scope”. To address the incompleteness, we proposed an integrative approach of measurement and simulation, using a combination of agent-based simulation and particle filter techniques for data assimilation.

As an evaluation of our proposed method, we conducted data assimilation based on pseudo-observational data and estimated transition tendencies of people in a virtual commercial facility. The measurement data were based on inflow count data from sensors installed in each store, and we tested whether the proposed method could compensate for the three identified incompleteness of the measurement techniques. The results indicated that estimating transition tendencies between two points using only inflow count data is difficult due to the high level of uncertainty involved. However, the estimation accuracy improves when attribute information is incorporated with the inflow count data. This indicates sensors such as cameras have potential usage for combining attribute information and roaming tendencies of people. Furthermore, transition tendencies between two or three points can be estimated using a combination of sample of actual transition sequence data and inflow count data, even when the samples are biased. This indicates that distributed inflow count data can be modified using biased sampling path data obtained from sensors such as Bluetooth/Wi-Fi. The insights gained from this study are crucial for future efforts to gather people flow data, which is often challenging to capture solely through conventional measurement. Future work will focus on evaluating the dynamic capabilities of the system using real-world data.
